# Ex Vivo Organ Perfusion Systems for Disease Modeling and Therapeutic Applications in Small Animal Models

**DOI:** 10.1155/term/2583925

**Published:** 2025-09-18

**Authors:** Mina Yeganeh, Andea Zito, Marwa Sadat, Agostino Pierro, Ian M. Rogers

**Affiliations:** ^1^Translational Medicine Program, Division of General and Thoracic Surgery, The Hospital for Sick Children, Toronto, Ontario, Canada; ^2^Institute of Medical Science, University of Toronto, Toronto, Ontario, Canada; ^3^Lunenfeld-Tanenbaum Research Institute, Sinai Health System, Toronto, Ontario, Canada; ^4^Department of Obstetrics & Gynecology, University of Toronto, Toronto, Ontario, Canada

## Abstract

Ex vivo organ perfusion (EVOP) is used for whole organ preservation, and the main focus is to improve the outcome of donor organs for transplantation. Recently, EVOP has found application in disease modeling, drug development, and tissue regeneration. We discuss progress in EVOP research involving small animal organs using benchtop and incubator-based EVOP systems, highlighting innovative designs of EVOP systems, technical specifications of each system, and their versatile applications across a range of research fields.

## 1. Introduction

Ex vivo organ perfusion (EVOP) systems aim to preserve whole organs outside of the body by maintaining them in a temperature-controlled environment and perfusing a solution through the vasculature. Over the past six decades, ex vivo whole-organ perfusion has undergone significant evolution from early exploration in research labs to its current clinical use for organ preservation prior to transplantation. One of the first EVOP systems was developed by Carrel and Lindbergh in 1935 to study the nutritional needs of organs including the thyroid, suprarenal gland, ovary, spleen, heart, and kidney [1]. It incorporated constant perfusion with a range of perfusion solutions, sterile conditions, and constant temperature to achieve optimal organ health [1]. Carrel and Lindbergh's work laid the foundation for future ex vivo perfusion research which further progressed in the 1960s, in parallel with advancements in surgical techniques used in organ transplantation.

Today, EVOP is used in transplant organ preservation [2, 3], expanding the pool of transplantable organs and reducing the donor organ shortage. For decades, EVOP research has relied mainly on large animal models for improving organ transplantation [4]. Compared to rodent models, porcine models have a greater similarity to humans in their genome sequence and immune system which are imperative when studying allogenic transplantation [4]. Their large size also facilitates easier cannulation of blood vessels and execution of transplant procedures [5]. While porcine models serve as ideal platforms for studying organ transplantation, their use in other applications—such as disease modeling, drug development, and tissue repair—remain constrained, largely due to high costs and limited availability. Small animal organs are more accessible, more cost-effective, and amenable to high-throughput modeling [5]. The widespread availability of genetically engineered models in rodents, especially in mice, also makes them better suited for disease modeling, compared to large animals. Therefore, small animal organs are valuable models for studying diseases, evaluating therapeutics, and overall advancing scientific pursuits.

## 2. EVOP in Small Animal Models

Scientific investigation has relied on diverse in vitro, in vivo, and ex vivo platforms, each offering unique benefits. In vitro cellular models are user-friendly, scalable for high-throughput assays [6], and can serve as patient-specific models [7]. Nevertheless, they fail to fully replicate organ physiology due to the absence of cellular heterogeneity and proper structural organization [8]. Conversely, in vivo animal models can represent human physiology; however, the complexity of these models poses challenges such as real-time visualization and assessment which require invasive procedures [5]. To address some of these challenges, scientists have developed three-dimensional multicellular *in vitro*–based models such as organoids and organ-on-chip devices to introduce more complexity to conventional cell cultures while maintaining controlled conditions for easier manipulation [9]. While organoids represent a more heterogeneous cell population, they do not contain all tissue layers and cell types [10]. Most organoid models also represent a fetal stage, making them less ideal for studying adult diseases [5]. Organ-on-chip devices also face challenges as most have been designed without taking into account the appropriate size and flow rate of each organ or full cell composition [11]. Other complex multicellular in vitro models such as tissue explants and organ slices contain the microscopic anatomical features specific to organs. However, their viability in culture is limited to shorter durations and the culture conditions do not accurately mimic physiological environments. EVOP models can address these shortcomings by mimicking physiologic conditions more accurately, while reducing the complexity of in vivo models [9]. These perfusion systems can isolate individual organs making it easier to study specific biological processes without systemic effects [5]. Compared to in vivo models, EVOP models are less costly and less time-consuming [5]. They also allow for real-time sampling of perfusion solutions for monitoring organ health [5]. Overall, EVOP systems represent isolated systems that closely recapitulate the cellular and the extracellular components of a whole organ. Owing to these benefits, EVOP platforms have gained much attention in a wide range of applications including disease modeling, cell therapy, drug testing, and tissue regeneration.

This review aims to explore the literature on EVOP systems designed for gut, lungs, kidneys, and pancreas, in small animals, focusing on applications excluding transplantation.

## 3. EVOP System Components

EVOP systems are designed to replicate physiologic conditions in order to sustain the viability of an organ ex vivo. To achieve dynamic flow, EVOP systems are often equipped with an inlet for antegrade perfusion of the organ, as well as an outlet for drainage of the perfusate solution. A peristaltic pump is usually used to achieve this dynamic flow. Perfusion systems can be designed to adjust for varying physiological measurements such as (i) oxygenation, (ii) flow dynamics, and (iii) temperature. Oxygenation in EVOP systems is important to maintain [12]. In addition to tubing material, the diameter of the tubing is important to the flow rate of the perfused organ. Flow rate contributes to the nutrient and oxygen supply of the tissue. Optimal flow rate is crucial to help meet the demands of the perfused tissue. To control for the temperature, some EVOP systems are kept in a temperature-controlled incubator. For benchtop EVOP systems, temperature can be manipulated by hot plates or circulating water around the tissue and perfusion media chambers.

## 4. Perfusion Solutions

There are several types of perfusion solutions that are commonly used in EVOP in both small and large animals. Perfusion solutions mimic physiological conditions necessary for organ function. These solutions typically contain: (a) electrolytes to maintain cellular ionic balance, (b) nutrients such as vitamins and amino acids to support organ function, (c) buffers to maintain desired pH levels (in the absence of CO_2_), (d) antibiotics to prevent contamination, and (e) glucose to provide a source of energy, and may include other supplements such as oxygen carriers, knockout serum or fetal bovine serum (FBS) [13]. One of the most common perfusion solutions is the Krebs–Henseleit solution [14]. Krebs solution contains calcium chloride, potassium chloride, sodium chloride, as well as magnesium sulfate, glucose, and sodium bicarbonate buffer or HEPES [14, 15]. Generally, if the perfusion system is set up inside a tissue-culture incubator with a CO_2_ source, sodium bicarbonate and HEPES buffers are not required as a pH buffer. However, in the absence of CO_2_—for instance, in most benchtop EVOP systems—these buffers are required. STEEN, which is most commonly used for perfusion of lungs in large animals and human organs, has recently been applied to kidney perfusion as well [16]. STEEN solution contains human serum albumin which maintains osmotic pressure, as well as dextran 40 which inhibits leukocyte adhesion to the endothelium [17, 18]. Both serum albumin and dextran 40 also maintain optimal oncotic pressure to prevent edema [18]. Waymouth's media is another commonly used perfusate in preclinical EVOP. Waymouth's media is a serum-free medium that contains essential amino acids, vitamins, salts, and glucose and is commonly used in liver perfusion [19, 20]. In the context of kidneys, renal epithelial cell (REC) media has been used for ex vivo kidney perfusion in small animals [5]. REC contains several growth factors that enhance kidney viability and allow long-term perfusion in preclinical models. From a clinical perspective, Ringer's lactate—an isotonic salt solution—has been used for ex vivo perfusion of discarded human kidneys, demonstrating clinical feasibility. Ringer's solution lacks the necessary nutrients and growth factors, making it suitable for clinical EVOP with shorter perfusion times, but not for preclinical models that require long-term perfusion [21].

Oxygenation can be achieved by several means: carbogen gas, red blood cells (RBCs), hemoglobin-based oxygen carriers (HBOCs) which contain hemoglobin in a cell-free suspension, or whole blood perfusate [22, 23]. Although effective, the utility of RBCs is hampered by their limited availability, high costs, short shelf life, and risks of infection transmission and hemolysis [22]. Synthetic HBOCs, however, are more accessible, have reduced risks of infections, and provide greater oxygenation compared to RBCs [24]. Nevertheless, studies have shown that HBOCs can react to nitric oxide, leading to hypertension and vasoconstriction [24]. Further investigation is warranted to advance the clinical application of HBOCs. Alternatively, whole blood perfusate can be used due to its high concentration of RBCs. When compared to RBCs and HBOCs, whole blood perfusate was observed to be superior in tissue preservation [25]. However, whole blood perfusates contain leukocytes which can be implicated in ischemia-reperfusion (I/R) injury of the tissue [26]. Leukocyte filtering improves tissue function and reduces cell death.

Overall, advancement in EVOP systems happens in parallel to optimization, refinement, and customization of perfusion solutions. The primary goal of the perfusate is to enhance preservation and function of organs ex vivo in order to prolong the duration of experimental procedures.

## 5. Small Animal EVOP Systems

### 5.1. Lung

Numerous studies have applied EVOP for lung disease modeling purposes (Table 1). EVOP has been used extensively to model pulmonary I/R injury in small animals. Maxey et al. used an isolated perfused lung model to study the effects of tumor necrosis factor-alpha (TNFα) production by resident lung cells in pulmonary I/R [27]. Harvested lungs from wildtype and TNFα-deficient mice were cannulated at the inferior vena cava and trachea for ex vivo media perfusion and ventilation, respectively [27]. The organs were kept within a benchtop bioreactor by Hugo Sachs Elektronik-Harvard Apparatus for a total of 120 min [27]. To induce I/R injury, perfusion was arrested and ventilation was maintained at hypoxic conditions [27]. After 60 min of ischemia, the organ was reperfused with Krebs solution and ventilation was returned to room air. Using this platform, they analyzed lung weight, lung injury score, pulmonary arterial and venous pressures, and pulmonary compliance (the ability of lungs to expand) [27]. The authors found that, upon exposure to I/R, perfused lungs from TNFα-deficient mice showed significantly lower histologic injury, reduced pulmonary edema and pulmonary artery pressure, and improved pulmonary compliance compared to wildtype [27]. This indicates that TNF-α released from resident lung cells is a key initiating factor in lung I/R injury. This ex vivo perfusion model allowed for continuous real-time monitoring of arterial and venous pressures during organ reperfusion providing valuable insights on progression of I/R injury. The model also allows researchers to study the role of lung-derived TNF-α in isolation, hence avoiding confounding factors from systemic proinflammatory responses that might contribute to pulmonary I/R injury.

**Figure 1:**
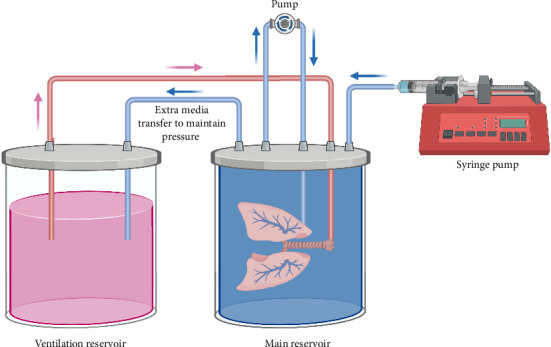
Graphical representation of a lung EVOP system developed by Ahmadipour et al. [28]. The system consists of two reservoirs, the main reservoir where the lung is perfused and the ventilation reservoir. The main reservoir is filled completely with media. A syringe pump continuously pumps media into the main reservoir, with any extra media flowing into the ventilation reservoir. The trachea is perfused from the ventilation reservoir media, while the pulmonary artery is perfused via a peristaltic pump that circulates the main reservoir media. The EVOP system was maintained in an incubator at 37°C (95% air; 5% CO_2_).

Several incubator-based EVOP systems have been developed to study the lungs in small animal models. In particular, in 2021, Ahmadipour et al. designed an EVOP system that achieved both vascular perfusion and liquid ventilation of mouse lungs via cannulation of pulmonary artery and trachea, respectively (Figure 1) [28]. They created a novel negative-pressure ventilation system that allowed control over tidal volume and achieved a physiological ventilation rate [28]. Using this platform, they studied the effect of higher ventilation rate (40 breaths per minute) on recellularization of mouse lungs. Decellularized mouse lungs were placed inside the EVOP system in a supine orientation (mimicking in vivo positioning) [28]. Lungs were then seeded with human bronchial epithelial cells and cultured for 3 days [28]. They showed that at higher ventilation rates compared to lower ventilation rates, lungs had an increased cell surface coverage, higher cell numbers, as well as significantly higher number of proliferative cells [28]. This innovative EVOP design allows for precise control of tidal volume and shows the importance of physiologically relevant mechanical cues for recellularization of lung grafts. Similar to this group, other researchers have addressed other important challenges in designing EVOP systems that mimic physiologic environments. Strict oxygen control is vital: hypoxia triggers apoptosis, whereas hyperoxia causes damage or dedifferentiation. This study introduced a tunable whole-lung bioreactor that adjusts oxygen delivery in real time to match metabolic demand. A calibrated mathematical model, validated with rat lungs, accurately predicted gas exchange, maintaining stable dissolved-oxygen levels, thus offering noninvasive insight into tissue metabolism [29].

Overall, there has been tremendous progress in ex vivo lung perfusion in both large animals, and more recently in small animal models. These models have been applied for studying various lung diseases including I/R injury and cancer, as well as for decellularization and tissue engineering purposes. Technical innovations have led to customizable EVOP platforms and data acquisition systems that support lung mechanics and provide high-level hemodynamic metrics. These modern systems offer real-time vascular pressure tracing, monitor respiratory rates, allow for easy assessment of gas exchange and injury biomarkers, and overall allow for highly controllable and reproducible perfusion circuits [30, 31]. Another state-of-the-art advancement has been the recent development of a low-volume *ex situ* lung perfusion (ESLP) system. This platform enables both single- and double-lung applications and requires only 17 mL of perfusate solution [32] within the circuit. This system not only reduces the amount and cost of perfusate significantly but also minimizes the number of animals needed. Although ex vivo lung perfusion research in small animals has made significant advancements, several critical gaps still remain. Currently, one of the major limitations in EVOP systems is the short perfusion times. Most described systems in rodent models can support up to 4–5 h of perfusion without damage to organ viability. In large animal models, this has been extended to 12 h. Extended perfusion times are needed for more accurate disease modeling purposes as well as therapeutic interventions. Another challenge of ex vivo lung perfusion in small animals is higher risk of atelectasis. Rodents have smaller airways, more delicate lung structures, and greater surface tension all of which make them more susceptible to atelectasis [33, 34]. To combat this, more precise ventilatory support is needed during the perfusion.

We believe that future work on ex vivo lung perfusion systems in small animals should be aimed at optimizing perfusate solutions and standardizing perfusion protocols. Currently, research on acellular and cellular-based perfusate solutions is more advanced in a clinical context compared to a preclinical small animal setting. In addition, there is a need to establish a consistent approach for ex vivo lung perfusion in small animals. This can ensure reproducibility and consistency within the field.

### 5.2. Kidneys

Ex vivo perfusion of kidneys has a long history spanning several decades (Table 2). In 1967, Nishiitsutsuji-Uwo et al. developed an isolated perfused rat kidney model based on previously published perfused models of rat liver [35, 36]. Using this system, they studied the metabolic activity of kidneys and found that under normal physiological levels, both glucose consumption and acetoacetate oxidation are important sources of fuel for basic respiration but not for secretion [35, 36]. They also discovered that glutamine production remained linear despite the decline of other additional products, such as aspartate, which is formed from the same precursor, glutamate [35, 36]. This demonstrated that the kidneys were an important source of the body's glutamine. The same perfusion apparatus was then adapted by Ross et al. in 1973 for their study of sodium reabsorption in an isolated rat kidney, specifically examining the preferred energy source for this process [37]. They optimized the perfusion media, enabling improved sodium reabsorption and glomerular filtration rate—markers of kidney functionality—that more closely resemble in vivo conditions [37]. It was found that glucose, not fatty acids, is the preferred energy source for sodium reabsorption [37]. Later in 2008, Rosenberger et al. adapted the same EVOP system to study acute kidney injury in diabetic rats [38]. Diabetic kidneys exhibited impaired function under hypoxic stress induced by the perfusion system, compared to the control [38]. This was evidenced by decreased glomerular filtration rate and sodium reabsorption, along with increased potassium secretion [38]. Additionally, there was extensive renal morphology damage, including severe tubular necrosis and collecting duct swelling [38]. Although functional impairment was observed in the perfusion system, it was not shown in vivo, as diabetic kidneys showed resilience to induced hypoxic stress. This phenomenon may be explained by a hypoxia-adaptation mechanism in the kidneys. The EVOP system allowed for this observation by inducing extreme hypoxic stress and preventing activation of this mechanism, which was difficult to achieve in vivo as it resulted in a high mortality.

**Figure 2:**
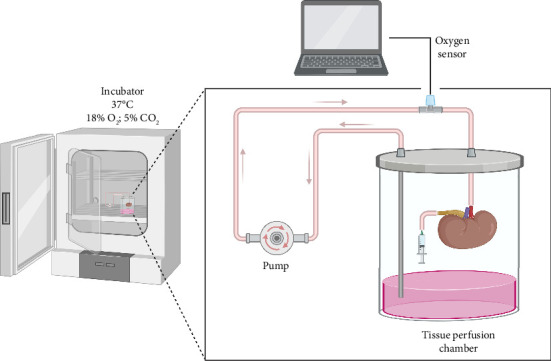
Graphical representation of an incubator kidney EVOP system developed by Won et al. [5]. The tissue perfusion chamber was connected to a closed perfusion loop. Media was pumped through the renal artery of mouse kidneys using a peristaltic pump. The renal vein drained directly into the media. An oxygen sensor was incorporated into the perfusion loop before the renal artery cannulation. The ureter was cannulated, and urine was collected via a syringe on day 7. The EVOP system was inside an incubator that maintained the temperature and oxygenation of the media.

An ex vivo kidney perfusion apparatus was designed and built by Won et al. in 2022 using mathematical modeling (Figure 2) [5]. Using a complementary error function and Henry's law, they predicted oxygen flux in the perfusion system and subsequently calculated the height of the organ chamber, the length of silicone tubing, and the flow rate required to maintain sufficient oxygenation of the kidney through atmospheric air [5]. The authors carefully developed a model that optimized surface area of the medium for sufficient oxygenation within the media reservoir [5]. Based on their calculations, they then fabricated an organ chamber containing a mouse kidney with the renal artery and the ureter both cannulated [5]. Kidneys were perfused with either DMEM +10% FBS or REC media for up to 9 days [5]. The experimentally measured oxygen levels of both arterial blood at the tissue level and at the vein exceeded the minimum physiological requirements, indicating that the mathematical modeling achieved the desired oxygen levels [5]. Urine analysis of total protein, albumin, and glucose levels relative to the concentrations found in the media indicated that perfused kidneys were properly functioning after 7 days. Low levels of total protein, albumin, and glucose were measured in the urine which implies that nutrients were absorbed, filtered by glomeruli, and reabsorbed by the proximal tubules [5]. This indicates accurate physiological processing of nutrients by the perfused kidneys. Furthermore, histology and immunofluorescence showed that kidneys remained healthy for up to 7 days ex vivo in the DMEM+10% FBS media and up to 9 days in the REC media [5].

Overall, kidney EVOP systems have undergone significant technical development. Small animal kidney EVOP systems have increased usage beyond organ transplantation. They are being utilized to investigate metabolic activity and nutrient source usage [35–37]. Furthermore, drug interactions [39] and hypoxic stress responses [38], difficult to study in vivo, have been observed using kidney EVOP systems. In comparison to other organ EVOP system's abilities to maintain tissue viability, small animal kidneys can be preserved for 7–9 days [5]. This ability expands the potential of disease modeling and mechanism investigations using kidney EVOP systems. However, this ability also highlights the technical aspects of EVOP systems important to optimizing the environment for the kidney. For example, continuous monitoring of perfusate flow rate in order to optimize oxygenation and meet the kidney's metabolic demand [5]. For future studies using kidney EVOP systems, adjustments to not only the perfusion media but also the monitoring systems may need to be made to ensure sufficient nutrient and oxygen supply to the tissue.

### 5.3. Gut

Gut ex vivo perfusion has been extensively documented (Table 3). In 2010, Lautenschlager et al. developed a perfused rat small bowel model to study the role of proinflammatory mediator platelet-activating factor (PAF) in intestinal edema [40]. Using a custom-made perfusion chamber from Hugo Sachs Elektronik-Harvard Apparatus, they assembled both vascular and luminal perfusion of the small bowel [40]. They demonstrated well-preserved gut viability after 4 h of perfusion by observing important functional parameters such as oxygen consumption, CO_2_ partial pressure, metabolism (lactate-to-pyruvate ratio), pH, cellular necrosis, and galactose uptake [40]. Additionally, intestinal morphology was preserved [40]. Administration of PAF led to vasoconstriction, inhibition of galactose uptake, as well as increased epithelial and endothelial permeability, which were reversed to normal levels after 30 min of perfusion [40]. Further, luminal effluent volume increased while the opposite was observed for vascular effluent volume. These findings emphasize the tightly controlled mechanisms of PAF in the intestine and the importance of luminal clearance as a defense mechanism. They also looked at underlying mechanisms of PAF-induced vasoconstriction and hyperpermeability and their involvement in intestinal failure [41].

**Figure 3:**
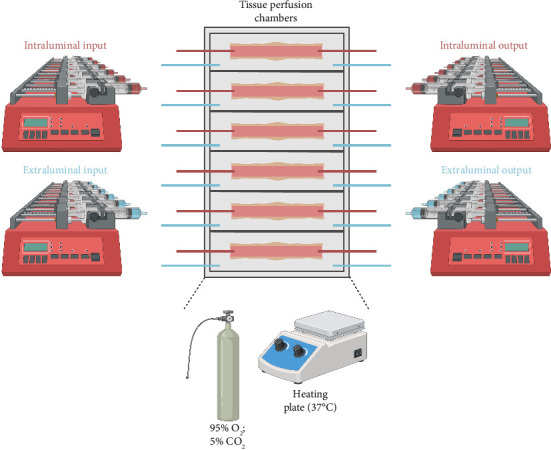
Graphical representation of a benchtop EVOP system developed by Yissachar et al. [42]. The tissue perfusion chamber consisted of six individual chambers for the cannulation of six intestines. Each intestinal chamber was connected to its own respective open perfusion loop. Media was perfused through the lumen and outside area of each individual intestine by synchronized intra- and extraluminal syringe pump inputs, respectively. The media was then withdrawn from synchronized intra- and extraluminal syringe pump outputs. The EVOP system was sealed and gassed using an oxygenator. Additionally, temperature was maintained using a heating plate.

In 2017, Yissachar et al. employed a silicone-based microfluidic chip that included six segments of mouse small intestine or colon (Figure 3) [42]. They utilized this perfusion system to evaluate intestinal immune and enteric nervous system (ENS) responses to bacteria. Tissue viability of the small intestine and colon was assessed through the analysis of morphology, mucus production, epithelial barrier integrity, proliferation, and enteric neurons [42].

Furthermore, they examined the immunocyte population present in the perfused tissues [42]. The viability of small intestine and colon was maintained for 2 and 24 h, respectively [42]. They then perfused germ-free immunocyte-activating bacteria intraluminally [42]. The perfusion system allowed them to observe immediate immune transcriptional changes in the small intestine and colon in response to bacteria. The ability to observe short-term transcriptional changes is not possible without the perfusion system, as the bacteria take days to fully develop within the gut in vivo. Additionally, they demonstrated that bacteria impact the ENS which subsequently affects immunocyte activation.

In addition to disease modeling, EVOP systems can be used to evaluate intestinal motility. Tan et al. evaluated the relationship between colonic distention (intraluminal pressure on intestinal tissue) and fatty acids (butyrate and propionate) and their role in induced intestinal motility in the isolated mouse colon [43]. They found that propionate reduced intestinal motility, but the effects were reversed upon increasing distention by adjusting the intraluminal pressure [43]. Intestinal motility increased proportional to distention. The same results were observed when butyrate was added after propionate, leading to enhanced intestinal motility [43]. However, this phenomenon of butyrate was observed at baseline distension. The effects of butyrate on intestinal motility were abolished in the presence of greater distension [43]. These findings highlight the importance of butyrate in cases of low intestinal motility that result from a fiber-deficient diet.

Several other groups have developed more compact ex vivo perfusion systems, complex enough to sustain organ function yet small enough to fit inside a standard tissue-culture incubator. Motherwell et al. designed a simple ex vivo model of rat intestinal mesenteric vasculature to study the effects of flow dynamics on angiogenesis [44]. After 48 h, static controls (no perfusion) had greater microvascular density compared to the perfused group. Additionally, invasive sprouting (into avascular regions) was greater in the perfused group while introverting (sprouting into central vascular regions) was greater in the static group [44]. These findings suggest that flow rate and shear stress greatly impact angiogenesis.

Similar to this model, Willi et al. developed a model of mouse intestinal mesentery by cannulating and perfusing a large mesenteric artery for 48 h [45]. They maintained viability after 48 h in 10% FBS-supplemented media but not in 1% or 5% FBS [45]. Despite the preserved tissue viability, there was significant reduction in microvasculature at 24 h and more so at 48 h [45]. Further analysis into this phenomenon revealed that microvasculature tortuosity remained high and constant over 48 h. This observation explains the preserved tissue viability, as tortuosity is a protection mechanism against increased intravascular pressure that could result in vascular damage.

In summary, intestinal EVOP systems are a useful platform that have been used to investigate molecular pathways41 and cellular interactions [42], as well as vasculature [44, 45] and motility dynamics [43] that are difficult to observe in vivo. Furthermore, they have been utilized to model diseases in a cost- and time-effective manner [46–48]. Despite the advancements of intestinal EVOPs, there are still limitations to their abilities due to the complex nature of the intestine. This complex nature involves interactions between the lumen, vasculature, ENS and muscular layer. To maintain these relationships ex vivo, bi-cannulation of both the vasculature and the lumen is important. Furthermore, the lumen contains bacteria and fungi that are essential to intestinal function in vivo. However, these microorganisms pose significant challenges to tissue viability ex vivo. To combat this, germ-free mice can be used but the microbiome–intestine crosstalk is lost. In addition to the overall complexity of the intestine, the small intestine and colon differ in both function and anatomy. This may add to the technical difficulties of maintaining intestinal tissue viability. For instance, one study was able to preserve the colon for 24 h, but only 2 h for the small intestine [42]. For future studies, the technical aspects of the EVOP system should be considered carefully for each component of the intestine. This includes the perfusion solution and flow dynamics that are respectively optimal for the small intestine, colon, and vasculature.

### 5.4. Pancreas

Ex vivo pancreas perfusion was first performed by Babkin and Starling in 1926 in a canine model using hypothermic machine perfusion [49, 50]. Many pancreas ex vivo perfusion studies followed (Table 4). Similar to other organs, early studies were not successful as they discovered several challenges during perfusion [51]. One major challenge is the fragile nature of the pancreas as it is a softer tissue organ and is composed mainly of exocrine glands that secrete digestive enzymes [52]. As a result, the pancreas is extremely sensitive to manipulation, and when injured, can secrete high levels of digestive enzymes (including protease and lipases) from its glands, leading to self-degradation of the tissue. Additional challenges seen in pancreas reperfusion include edema, hemorrhage, and venous congestion.

Various cold storage perfusates have been developed for the pancreas, with the most popular being the University of Wisconsin (UW) solution, which has become the gold standard preservation solution for the pancreas for 20 years [53]. The UW solution was created based on studies using the canine pancreas, where Wahlberg et al. demonstrated that this perfusate allowed for optimal control of edema allowing for preservation up to 24 h [54].

The first use of rodent pancreas in EVOP was performed by Loubatieres' group in 1980, using a Krebs–Ringer bicarbonate buffer including albumin and glucose, as well as bubbled oxygen and carbon dioxide [55]. They studied the effects of hypothermia on endocrine function of the pancreas by culturing the organ at normothermic (37.5°C) and hypothermic (28°C) temperatures. Insulin secretion was measured following stimulation by glucose, tolbutamide, and acetylcholine [55]. They found that under hypothermic temperatures, glucose-responsive insulin secretion was reduced, suggesting that normothermic perfusion is superior for maintaining normal endocrine function [55]. Shortly after, Pegg et al. generated a rat pancreas normothermic perfusion system named the “single-pass method” [56]. This system contained two separate circuits with different glucose concentrations, allowing for a switch between them without recirculation of the perfusate in order to easily assess insulin release upon changes in glucose concentration [56]. Pegg et al. demonstrated that the pancreas could be cultured for up to 2 h using their system with normal glycemic responsiveness and low levels of edema, as measured by wet-to-dry weight ratio of the tissue before and after perfusion [56]. They also demonstrated that mannitol, a slowly permeating neutral solute, resulted in improved preservation [56]. Furthermore, by adjusting the electrolyte balance (substituting half of the extracellular sodium ions with intracellular ions such as potassium and magnesium), they significantly improved the preservation solution [56]. This alteration boosted the baseline insulin secretion and improved the insulin response to glucose stimulation [56].

**Figure 4:**
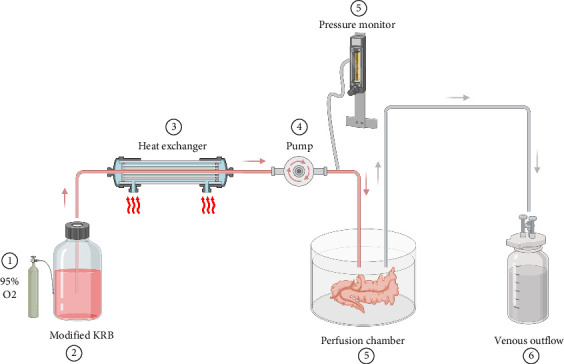
Graphical representation of a benchtop rat pancreas EVOP system developed by Mantke et al. [58]. The rat pancreas was isolated and cannulated at the caudal aorta (input) and portal vein (output) using Teflon tubing. The perfusate (modified Krebs solution) was gassed with 95% O_2_ and 5% CO_2_ and pumped at a constant flow rate while perfusion pressure was measured by a pressure transducer. A heat exchanger was used to maintain the perfusate at a constant temperature of 37°C. The perfusate was pumped into the cannulated caudal aorta, into the pancreas, and was pumped out of the pancreas through the portal vein, which flowed into a collection bottle, creating an open system.

Due to the specific challenges linked to ex vivo perfusion of the pancreas, particularly its sensitivity, the duration of ex vivo perfusion for rodent pancreas has been limited to a few hours. One study investigated the effects of leptin on endocrine function by measuring insulin secretion from the isolated pancreas [57]. They demonstrated that leptin does not regulate beta cell function, as the addition of leptin to the system had no effect on carbachol-induced insulin secretion [57]. Other studies measured secretion of pancreatic enzymes (amylase and lipase) in the perfusate, which are indicative of exocrine cell damage [58]. A graphical representation of their EVOP system is shown in Figure 4. In recent years, improvements have been made to pancreas EVOP systems to preserve the tissue for longer periods of time. Since the pancreas is a low vascularized organ and highly susceptible to edema when subjected to high pressures, low-pressure perfusion was introduced [59]. These rodent models contribute uniquely to the study of islet biology but improvements need to be made to extend the organ preservation time, as these studies were limited to short-term maintenance over a few hours.

Recent advances in ex vivo perfusion of pancreas in preclinical models have highlighted the importance of these systems not just for organ preservation and transplantation but also as platforms for disease modeling and studying mechanisms of therapeutic interventions. For instance, numerous studies have used rodent EVOP systems to investigate how the endocrine and exocrine functions of pancreas are impacted by certain conditions (i.e. hypothermia) or therapeutic interventions. These platforms can serve as valuable tools for type 1 diabetes research in assessing how drugs modulate glucose, lactate, and insulin release in physiologically relevant models. Despite progress in ex vivo pancreas perfusion, a major remaining challenge is the risk of edema. Due to its fragile microvasculature and high vascular permeability, the pancreas is highly sensitive to hydrostatic pressure changes [60, 61]. In large animal models, several studies have established low-pressure systems that achieve optimal oxygenation while maintaining tissue integrity. However, further refinement is needed in small animal EVOP models. To combat edema and extend pancreas viability during ex vivo perfusion, addition of a dialysis circuit would be beneficial as it helps remove metabolic waste [60, 62]. Future efforts should focus on developing and optimizing a dialysis circuit to enhance perfusion outcomes.

## 6. Discussion

Both benchtop and incubator-based EVOP systems have been used widely in small animals for various applications including disease modeling, evaluation of novel therapies, advancing our understanding of organ physiology, as well as regenerative medicine research. Compared to in vivo models, incubator-based and benchtop perfusion systems both allow for more precise control of pressure, oxygenation, and other experimental parameters, and offer real-time visualization of the perfused organ. Benchtop EVOP systems struggle to maintain sterile environments, hence increasing chances of contamination [5]. This significantly limits perfusion of organs for extended periods of time. However, incubator-based EVOP systems minimize the risks of contamination due to the sealed environment of the incubator. Incubator perfusion systems are also more cost-effective, easier to operate, and take up less space. However, both benchtop and incubator EVOP systems face challenges in maintaining physiologic support during prolonged periods of organ perfusion. To remain viable, organs require a continuous supply of nutrients and oxygen, along with constant removal of toxic metabolites. Lack of metabolic regulation in current EVOP systems leads to inflammation, cell death, and organ damage within a short period of time. To address this issue, several technologies have been proposed. For instance, integration of white blood cell filters within the circuit can block leukocyte infiltration, hence mitigating the inflammatory response [63, 64]. Studies show that integration of a cytokine filter during lung [65] and kidney [66] perfusion reduced overall proinflammatory cytokine expression, reduced edema formation, and improved blood flow. This technique has primarily been used in large animal models and humans [67]. While further development is needed for small animal models, it is important to consider the implications of leukocyte depletion on disease modeling and drug development. Another approach to supply the organ with sufficient nutrients and oxygen and remove metabolic waste is through period replacement of the perfusate within the circuit with fresh solution. This method requires large volumes of perfusate, making it expensive. To reduce costs, efforts have been made to recycle perfusate using dialysis-based systems [68]. Finally, xenogeneic cross-circulation is another technique used for enhancing physiologic support during EVOP. First developed by O'Neill et al. in 2017, this technique involves exchanging whole blood between an ex vivo organ and a living organism [69]. Cross-circulation of human donor lungs with swine models has not only extended duration of perfusion [69] but also improved gas exchange and dynamic compliance [70]. Similar to leukocyte filters, this method has only been explored in large animal models and discarded human organs, highlighting the need for further development in small animals.

While these technological advances aim to improve physiologic support during EVOP, system design should also be tailored to the specific functional and anatomical requirements of different organs. For instance, the gut contains both luminal (intestinal lumen) and vascular (blood vessels) perfusion. Luminal perfusion can be used to model intestinal absorption and secretion, as well as studying the epithelium while vascular perfusion is required for achieving sufficient nutrients and oxygen as well as studying the endothelium.

The ex vivo perfusion of lungs also requires additional considerations. To closely mimic physiologic conditions, lungs must be ventilated to simulate lung expansion and contraction [71]. This is especially important for studying normal lung physiology, evaluating therapeutic interventions, as well as preserving lungs for transplantation. To simulate the humid environment of the lungs, researchers have developed methods for wet ventilation which usually involves suspending the lung in media and connecting it to a ventilator [71]. In other cases, dry ventilation can be used to allow for air exchange in the lung, which is closer to physiological and a good environment for disease modeling purposes [46].

Additionally, the kidneys require only vasculature perfusion, but the ureter can be also cannulated to collect urine in order to monitor kidney function. Because of the density of the kidney, optimization of the perfusate flow rate and pressure is important to minimize physical damage but still maintain oxygen and nutrient delivery. The addition of a vasodilator has proven useful in porcine EVOP [72].

The pancreas presents unique considerations for EVOP due to the unique properties of the organ. Being a highly sensitive organ with low flow and pressure, it is difficult to achieve an optimal flow rate. Inadequate flow rates may result in insufficient perfusion, leading to cell death, while too high flow rates can quickly lead to edema and subsequent tissue damage, which has been a challenge for many groups. Furthermore, given its predominantly exocrine composition, the pancreas is particularly prone to the secretion of digestive enzymes, such as proteases and lipases, especially under stress or manipulation. Therefore, the inclusion of enzyme inhibitors, such as trypsin inhibitor, is crucial to prevent autodigestion of the organ.

## 7. Conclusions

In conclusion, EVOP systems in small animals have enabled researchers to study organ physiology, model complex disease mechanisms, and investigate novel therapeutic agents. EVOP systems in small animals represent a cost-effective yet physiologically relevant model that can significantly progress the field of organ perfusion. Here, we highlighted four organ systems. Although there are common ex vivo parameters that must be met for all organs, such as nutrients, flow rates, and oxygenation, specific organ adaptations such as ventilation for the lung, or electrical nerve stimulation for the heart must be considered. The heart EVOP has a long history that has led to the modern benchtop EVOP systems now used for multiple organs. Bowditch, in 1869, using a frog heart, connected to a cannula and reservoir of rabbit serum, isolated the vagus nerve from the spinal cord to the heart, and was able to stimulate it ex vivo. The Langendorff ex vivo heart system, developed in 1895, revealed the importance of oxygenation to heart function [73]. Modern systems are being used to regenerate acellular organs for organ replacement therapies and evaluate novel pharmaceutical treatments. These systems prove to be valuable models owing to their ability to replicate physiologic conditions in an ethical, cost-effective, and timely manner. Ultimately, small animal EVOP systems offer a powerful and practical platform for advancing medical research and therapeutic discovery.

## Figures and Tables

**Table 1:** Summary table listing all publications discussed in this manuscript on ex vivo lung perfusion.

Study	Animal species/strain	Perfusion solution	Tubing type/length	Use of oxygenators	Flow rate	Length of perfusion	Temperature (°C)	Application	Functional tests performed	Cannulation site
Lung										
Benchtop										
Maxey, 2004	C57BL/6 mice (adult males)	Krebs–Henseleit buffer containing 2% albumin, 0.1% glucose, and 0.3% HEPES (335–340mOsm/kg)	Not described	No	2 mL/min	60 min	37°C	Modeling pulmonary I/R injury	Lung weight, lung injury score, pulmonary artery and vein pressures, airway resistance, pulmonary compliance	Trachea for ventilation; inferior vena cava for media perfusion
Zhao, 2006	C57BL/6 mice (adult males)	Krebs–Henseleit buffer containing 2% albumin, 0.1% glucose, and 0.3% HEPES (335–340mOsm/kg)	Not described	No	60 μL/g body weight/min	60 min	37°C	Investigating the role of alveolar macrophages in pulmonary I/R injury	Lung weight, cytokine/chemokine expression (RT-PCR, ELISA), lung vascular permeability, neutrophil quantification, pulmonary artery pressure, pulmonary compliance	Trachea for ventilation; inferior vena cava for media perfusion
Francioli, 2017	Sprague–Dawley rat (300 g)	Steen® solution (Xvivo perfusion, Göteborg, Sweden, pH 7.4)	Not described	Gas-exchange membrane	Not described	4 h	37°C	Testing of pharmaceutical drug pyrrolidine dithiocarbamate (PDTC) during warm ischemia	Physiological variables (PA pressure, arterial pressure, vascular resistance, compliance), protein concentration and lactate dehydrogenase activity in bronchoalveolar lavage, pro-inflammatory cytokine levels, NF-kB expression	Pulmonary artery for media perfusion; trachea for ventilation
Wang, 2016	Sprague–Dawley rat (400 g)	Steen solution (Xvivo perfusion, Göteborg, Sweden, pH 7.4)	Not described	Gas-exchange membrane	Not described	3 h	37°C	Effect of MnTBAP and 3-AB on the lung during warm ischemic injury	Physiological variables (PA pressure, arterial pressure, vascular resistance, compliance), protein concentration and lactate dehydrogenase activity in bronchoalveolar lavage, pro-inflammatory cytokine levels, histology	Pulmonary artery for media perfusion; trachea for ventilation
Incubator										
Mishra, 2012	Sprague–Dawley rats (6-12-week-old male)	RPMI	Silicone (3 m)	No	6 mL/min	7–14 days	37°C	Modeling human lung cancer on a decellularized rat lung matrix	Histology, DNA extraction, immunohistochemistry	Trachea for cell seeding, pulmonary artery for media perfusion
Ahmadipour, 2021	C57BL/6 mice (12-14-week-old males)	High glucose content DMEM containing 10% FBS and 1% antibiotics	Not described	No	1.5 mL/min (vascular perfusion rate); 40 breaths/min with a tidal volume of 300 μL (ventilation rate)	3 days	37°C	Describing a novel ventilation protocol for enhanced recellularization of rat lung scaffolds	Histology, immunofluorescence staining, immunohistochemistry, RT-PCR	Trachea for liquid ventilation and cell seeding, pulmonary artery for media perfusion
Engler, 2018	Sprague–Dawley rat (200 g)	DMEM high glucose with 10% FBS, 100 U/mL penicillin, 100 μg/mL streptomycin, 3 μg/mL amphotericin B and 50 μg/mL gentamicin	Silicone (1.5 m)	Hollow fiber cartridge oxygenator	4 mL/min (perfusion rate); 4, 6, 8, 16, or 32 mL/min (oxygenator flow rate)	24 h	37°C	Mathematical modeling and characterization of oxygen requirements of whole lung tissues	Histology, immunofluorescence staining, whole lung cell count, total lung weight, single cell oxygen consumption rate, glucose consumption, lactate production	Pulmonary artery for media perfusion

**Table 2:** Summary table listing all publications discussed in this manuscript on ex vivo kidney perfusion.

Study	Animal species/strain	Perfusion solution	Tubing type/length	Use of oxygenators	Flow rate	Length of perfusion	Temperature (°C)	Application	Functional tests performed	Cannulation site
Kidney										
Benchtop										
Nishiitsutsuji-Uwo, 1967	Wistar rats (adult males)	Krebs–Henseleit saline, 4.5% bovine serum albumin	Portex based	Yes, but details not specified	16–30 mL/min	< 2 h (dependent on test)	38°C–40°C	Studying metabolic activity of the kidney	Metabolism, oxygen consumption, glucose synthesis, creatinine clearance, urine analysis	(Vascular) renal artery and vein; ureter
Ross, 1973	Wistar rats (adults)	Krebs–Henseleit high-bicarbonate saline, 10% bovine serum albumin (mixed with other solution dependent on test)	Not described	95% O2; 5% CO2	34.5 mL/min	> 4 h	Not described	Studying energy sources for sodium reabsorption	Physiological variables (metabolism, oxygen consumption, glucose synthesis, creatinine clearance, urine analysis, glomerular filtration rate), sodium absorption	(Vascular) renal artery and vein; ureter
Rosenberger, 2008	Sprague-Dawley rats (adult males)	Modified Krebs–Ringer–Henseleit solution, 6.7 g/dL bovine serum albumin, 20 amino acids	Not described	95% O2; 5% CO2	Not described	90 min	38°C	Studying acute kidney injury in diabetic rats	Physiological variables (creatinine clearance, plasma creatinine, urine analysis, sodium absorption, potassium excretion, glomerular filtration rate, necrosis), kidney morphology	(Vascular) renal artery and vein; ureter
Hori, 1993	Wistar rats (adult males)	Modified Krebs–Henseleit bicarbonate buffer, bovine erythrocytes,5% bovine serum albumin,8 amino acids,5 nM glucose,3% mannitol, and 100 μg/mL creatinine	Not described	95% O_2_; 5% CO_2_	5 mL/min	15–30 min	Not described	Studying mechanism of digoxin excretion	Physiological variables (perfusate flow rate, urine flow rate, glomerular filtration rate, sodium and glucose fractional reabsorption), renal tubular secretion	(Vascular) renal artery and inferior vena cava; ureter
Yokota, 2018	Wistar rats (12 week old males)	Modified Krebs–Henseleit solution ,6 g% bovine serum albumin, 8.3 mM urea, 1.25 mM inulin, and 5.55 mM glucose	Not described	Silastic membrane oxygenator	20 mL/min	90 min	37°C	Studying the metabolic effects of high fructose intake	Renal perfusion pressure, renal vascular resistance, osmolar clearance, glomerular filtration rate, urinary flow	(Vascular) renal artery and vein; ureter
Mahboub, 2020	Lewis rats (adult males)	Williams medium E containing 1000 U/L heparin, 15% albumin, 1000 µmol/L creatinine, and 25% HBOC-201	Silicone based	95% O_2_; 5% CO_2_	≥ 8 mL/min (to maintain physiological pressure)	90 min (rewarming) then 120 min (reperfusion)	10°C–37°C	Studying the effects of hemoglobin based oxygen carriers during gradual kidney rewarming	Physiological variables (ultrafiltrate production, glomerular filtration rate, sodium reabsorption, pH, lactate levels), renal artery resistance, weight, oxygen consumption, energy, kidney morphology	(Vascular) renal artery; ureter
Incubator										
Uzarski, 2015	Sprague-Dawley rats (adult males)	(Decellularization) Triton X-100, sodium dodecyl sulfate solution; (recellularization) DMEM/F-12, 10% FBS, 1% penicillin/streptomycin	Silicone based	5% CO2	4 mL/min	7 days	37°C	Studying the effects of seeding canine or human derived renal cells onto decellularized rats kidney	Hydrostatic pressure, metabolic activity, kidney morphology, injury (kidney injury molecule 1)	(Vascular) renal artery; ureter
Won, 2022	CD1 mice (adults)	(Media 1) Dulbecco's modified Eagle's medium, 10% fetal bovine serum; (media 2); renal epithelial cell media	Silicone based	18.6% O2; 5% CO2	1.1 mL/min	9 days	37°C	Mathematically designed EVOP system for improving kidney longevity	Oxygen consumption, urine analysis, kidney morphology, immunostaining	(Vascular) renal artery; ureter

**Table 3:** Summary table listing all publications discussed in this manuscript on ex vivo gut perfusion.

Study	Animal species/strain	Perfusion solution	Tubing type/length	Use of oxygenators	Flow rate	Length of perfusion	Temperature (°C)	Application	Functional tests performed	Cannulation site
Gut										
Benchtop										
Lautenschlager, 2010	Wistar rats (adult females)	(Vascular) modified Krebs–Henseleit solution; (luminal) 114 mM NaCl, 5 mM KCl, 26 mM NaHCO_3_, 30 mM lactose, 5.55 mM glucose, 10 mM mannitol, and 0.8 mM glutamine (310–320 mOsm/L)	Tygon based	95% O_2_; 5% CO_2_	(Vascular) 7.5 mL/min; (luminal) 0.15 mL/min	240 min	36°C	Studying the role of platelet-activating factor in intestinal edema	Physiological variables (oxygen consumption, CO_2_ partial pressure, metabolism, pH, cellular necrosis, galactose uptake, mucosal surface scoring), luminal and vessel fluid measurements, mesenteric artery and vein pressure, luminal pressure, intestinal motility	(Vascular) superior mesenteric artery and hepatic portal vein; (luminal) proximal and distal small intestine
Boyle, 2016	Wistar rats (adult females)	(Vascular) modified Krebs–Henseleit solution; (luminal) 114 mM NaCl, 5 mM KCl, 26 mM NaHCO_3_, 30 mM lactose, 5.55 mM glucose, 10 mM mannitol, and 0.8 mM glutamine (310–330 mOsm/L)	Tygon based	95% O_2_; 5% CO_2_	(Vascular) 7.5 mL/min; (luminal) 0.05 mL/min	240 min	37°C	Modeling Salmonella enteritis	Intestinal epithelial scoring, epithelial tight junction staining, pro-inflammatory cytokine levels, arterial pressure, intestinal absorption (galactose uptake), intestinal lumen fluid analysis	(Vascular) superior mesenteric artery and hepatic portal vein; (luminal) proximal and distal small intestine
Gagliardi, 2021	Balb/c mice (8 weeks)	IMDM containing 20% Knockout serum replacement, 2% B-27,1% of N-2,1% L-glutamine, 1% non-essential amino acids, 1% HEPES	Not described	95% O_2_; 5% CO_2_	99 μL/hr	16 h	37°C	Modeling celiac disease	MTT assay, unfolded protein response levels, gliadin derived peptides, intestinal permeability, pro-inflammatory cytokine levels, intestinal morphology	Proximal and distal small intestine
Gagliardi, 2021	Balb/c mice (13 days)	IMDM containing 20% Knockout serum replacement, 2% B-27,1% of N-2,1% L-glutamine, 1% non-essential amino acids, 1% HEPES	Not described	95% O_2_; 5% CO_2_	99 μL/h	5 h	37°C	Modeling inflammatory bowel disease	MTT assay, unfolded protein response levels, intestinal permeability, pro-inflammatory cytokine levels, intestinal morphology	Proximal and distal colon
Tan, 2020	C57BL/6 mice (8–10 week old females)	(Extraluminal) modified Krebs solution; (intraluminal) Phosphate buffer solution	Not described	95% O_2_; 5% CO_2_	30 μL/min	> 30 min (dependent on test)	35°C	Studying the effects of distention and fatty acids on intestinal motility	Colonic motor complex activity, diameter of colon, intraluminal pressure and pressure amplitude, pellet expulsion monitoring	Proximal and distal colon
Schreiber, 2014	Sprague-Dawley rats (adult)	(Vascular) Tyrode solution; (luminal) Tyrode solution	Not described	95% O_2_; 5% CO_2_	(Vascular) 3 mL/min; (luminal) 80 mL/h	> 30 min (dependent on test)	37°C	Pharmacological testing and effects on intestinal motility	Jejunum intestinal motility, intestinal morphology	(Vascular) superior mesenteric artery and vein; (luminal) proximal and distal jejunal region
Incubator										
Motherwell, 2019	Wistar rats (adult males)	Culture media, 10% fetal bovine serum	Not described	5% CO_2_	0.12 mL/min	48 h	37°C	Studying the effects of flow dynamics on angiogenesis	Vascular analysis (immunofluorescent imaging, diameter, velocity, shear stress, sprouting)	Main feeding artery and vein
Willi, 2022	NG2DsRedBAC mice (6–12 months); C57BL/6 mice (6–12 months)	Minimum essential media, 1,5, or 10% fetal bovine serum, 1% penicillin-streptomycin	Silicone based	5% CO_2_	1–1.5 mL/min	48 h	37°C	Studying viability and dynamics of intestinal vasculature	Vascular analysis (immunofluorescent imaging, density tortuosity), cell viability	Feeder artery

**Table 4:** Summary table listing all publications discussed in this manuscript on ex vivo pancreas perfusion.

Study	Animal species/strain	Perfusion solution	Tubing type/length	Use of oxygenators	Flow rate	Length of perfusion	Temperature (°C)	Application	Functional tests performed	Cannulation site
Pancreas										
Benchtop/incubator										
Single-pass method (Pegg, 1982)	WAG rats	Gelatin polypeptide Haemaccel + 5.4 mM calcium + glucose (5 mM for first hour, 30 mM for second hour)	Not described	Bubbled oxygen (95% O_2_, 5% CO_2_)	2 mL/min	2 h	38°C	To test assays for measuring glucose-responsiveness	Insulin secretion assay (5 mM vs. 30 mM glucose)	Input: Aorta (below superior mesenteric artery)
Recirculation method (Pegg, 1982)	WAG rats	Gelatin polypeptide Haemaccel + 5.4 mM calcium + 5 mM glucose	Not described	Bubbled oxygen (95% O_2_, 5% CO_2_)	60 mmHg (pressure)	2 h	38°C	To test assays for measuring glucose-responsiveness	Insulin secretion assay (5 mM vs. 30 mM glucose)	Input: Aorta (below superior mesenteric artery)
Loubatieres-Mariani, 1980	Wistar rats	Krebs–Ringer bicarbonate buffer + 2 g/L purified bovine albumin + 0–3 g/L glucose	Not described	Bubbled oxygen (93% O_2_, 7% CO_2_)	2.4 mL/min	90 min	28°C & 37.5°C	To test effect of temperature (hypothermia) on glucose responsiveness	Insulin secretion assay (following stimulation with high glucose, tolbutamide, acetylcholine, and glucagon)	N/A
Kojima, 1984	Wistar rats	Na+; 131 mEq/1, lactate-: 28 mEq/L, K+: 4 mEq/1, Ca∗: 3 mEq/L, CI-: 110 mEq/1, Dextran 40:30.0 gr/1, glucose: 1 gr/1, pH: 8.2 + 0.2. T	Not described	Artificial lung	10 mL/min	6 h	4°C (hypothermic)	To test the effect of hypothermia, hyperbaria, and oxygenation during pancreas preservation on islet health and function	Insulin secretion assay; transplantation into diabetic mice for reversal of diabetes	Input: celiac axis
Klempanauer, 1982	Wistar rats	Protide gel solution, fructose-bicarbonate solution, modified cryoprecipitated plasma, Collins' C3 solution, Sack's solution	Not described	No	Not described	24 h	0°C–4°C	To demonstrate that a simpler approach, using initial perfusion with a chilled solution, following by storage at 0–4C is superior to other methods for 24 h perfusion	N/A	Input: Aorta (below superior mesenteric artery)
Mantke, 2009	Wistar rats	Modified Krebs–Ringer bicarbonate	Not described	95% O_2_, 5% CO_2_	1 mL/min (220 mmHg)	80 min (60 min perfusion time, 20 min equilibration time)	37°C (using heat exchanger)	To examine early mechanisms leading to acinar cell injury in acute pancreatitis	Amylase, lipase, and lactate dehydrogenase activity	Causal aorta (input), portal vein (output). Both cannulated with Teflon tube

## Data Availability

Data sharing is not applicable to this article as no datasets were generated or analyzed during the current study.
